# The Effect of Gut-Training and Feeding-Challenge on Markers of Gastrointestinal Status in Response to Endurance Exercise: A Systematic Literature Review

**DOI:** 10.1007/s40279-023-01841-0

**Published:** 2023-04-15

**Authors:** Isabel G. Martinez, Alice S. Mika, Jessica R. Biesiekierski, Ricardo J. S. Costa

**Affiliations:** Department of Nutrition, Dietetics and Food, Monash University, Level 1, 264 Ferntree Gully Road, Notting Hill, VIC 3168 Australia

## Abstract

**Background:**

Nutrition during exercise is vital in sustaining prolonged activity and enhancing athletic performance; however, exercise-induced gastrointestinal syndrome (EIGS) and exercise-associated gastrointestinal symptoms (Ex-GIS) are common issues among endurance athletes. Despite this, there has been no systematic assessment of existing trials that examine the impact of repetitive exposure of the gastrointestinal tract to nutrients before and/or during exercise on gastrointestinal integrity, function, and/or symptoms.

**Objective:**

This systematic literature review aimed to identify and synthesize research that has investigated the impact of ‘*gut-training*’ or ‘*feeding-challenge*’ before and/or during exercise on markers of gastrointestinal integrity, function, and symptoms.

**Methods:**

Five databases (Ovid MEDLINE, EMBASE, CINAHL Plus, Web of Science Core Collection, and SPORTDiscus) were searched for literature that focused on gut-training or feeding-challenge before and/or during exercise that included EIGS and Ex-GIS variables. Quality assessment was conducted in duplicate and independently using the Cochrane Collaboration’s risk-of-bias (RoB 2) tool.

**Results:**

Overall, 304 studies were identified, and eight studies were included after screening. Gut-training or feeding-challenge interventions included provision of carbohydrates only (*n* = 7) in various forms (e.g., gels or liquid solutions) during cycling or running, or carbohydrate with protein (*n* = 1) during intermittent exercise, over a varied duration (4–28 days). Gut discomfort decreased by an average of 47% and 26% with a 2-week repetitive carbohydrate feeding protocol (*n* = 2) and through repeated fluid ingestion over five trials (*n* = 1), respectively. Repetitive carbohydrate feeding during exercise for 2 weeks resulted in the reduction of carbohydrate malabsorption by 45–54% (*n* = 2), but also led to no significant change (*n* = 1). The effect of gut-training and feeding-challenges on the incidence and severity of Ex-GIS were assessed using different tools (*n* = 6). Significant improvements in total, upper, and lower gastrointestinal symptoms were observed (*n* = 2), as well as unclear results (*n* = 4). No significant changes in gastric emptying rate (*n* = 2), or markers of intestinal injury and permeability were found (*n* = 3). Inconclusive results were found in studies that investigated plasma inflammatory cytokine concentration in response to exercise with increased carbohydrate feeding (*n* = 2).

**Conclusions:**

Overall, gut-training or feeding-challenge around exercise may provide advantages in reducing gut discomfort, and potentially improve carbohydrate malabsorption and Ex-GIS, which may have exercise performance implications.

**Supplementary Information:**

The online version contains supplementary material available at 10.1007/s40279-023-01841-0.

## Key Points

**Table:** 

Repetitive exposure to nutrition before and during exercise can help train the gastrointestinal tract, and subsequently improve gastrointestinal function, feeding tolerance, and reduce incidence and severity of exercise-associated gastrointestinal symptoms (Ex-GIS), leading to potential exercise performance benefits.
‘*Gut-training*’ and ‘*feeding-challenge*’ before and/or during exercise reduces gut discomfort, upper Ex-GIS, and carbohydrate malabsorption, but the effect on other gastrointestinal functional responses, gastrointestinal integrity, and systemic responses remains unclear and elusive.
Improvements in carbohydrate malabsorption and reduction in Ex-GIS may lead to better exercise performance.
Future research requires the use of more sensitive and specific tools (i.e., non-metabolizable sugar probes, modified visual analog scale [mVAS], etc.) in assessing gastrointestinal status to elucidate underlying mechanisms which would help fine tune gut-training guidelines and recommendations.

## Introduction

Exogenous carbohydrate supplementation during prolonged exercise is known to delay fatigue onset and support performance, and is subsequently well established in international guidelines and consensus statements [1, 2]. Previous carbohydrate guidelines and recommendations for endurance exercise up to 2 h and > 3 h are suggested at 30–60 g/h and up to 90 g/h, respectively, with multi-transportable carbohydrates (2:1 glucose to fructose ratio) preferred [2]. These recommendations have recently been challenged with respect to tolerance—both in the gastrointestinal and feeding aspects, especially in ultra-endurance exercise settings, which has led to changes in guidelines and recommendations [1, 3]. As an example, the rate of carbohydrate use depends largely on intensity, which tends to be lower in longer races; thus, current recommendations state targeting 30–90 g/h of carbohydrates depending on needs and other factors such as tolerance and practicality [1, 3–6]. Given that absolute exercise intensity tends to be lower in ultra-endurance events, and if gastrointestinal issues are a concern, then caution must be taken with higher intake rates. Trialing and practicing nutrition strategies, including the form, composition, quantity, and quality of food to be ingested, would be best practice [3]. Nevertheless, multi-transportable carbohydrate recommendations have been shown to enhance endurance exercise performance, with improvements proportional to carbohydrate intake levels and tolerance [7, 8]. Aside from maintaining a high circulating glucose availability for potential use in intramuscular glycolysis and oxidative phosphorylation, a greater reduction in markers of intestinal epithelial injury and small intestinal permeability was observed with glucose ingestion compared with water during 2 h of running [9]. Field studies of various endurance and ultra-endurance athletes such as long-distance runners, cyclists, and triathletes [10–16] have shown that these target intakes are challenging to achieve. This could be caused by appetite suppression, gastrointestinal discomfort, and exercise-associated gastrointestinal symptoms (Ex-GIS) onset; and individual feeding tolerance level during exercise, likely associated with exercise-induced gastrointestinal syndrome (EIGS) [17]. Individualized rate-limiting factors also include intake behavior, gastrointestinal functional responses, blood glucose availability, and skeletal muscle glucose uptake and oxidative metabolic pathways [18]. Aside from an athlete’s physiological ability to tolerate feeding during exercise, it is also worthwhile to consider other factors such as motivation (e.g., willingness to employ a nutrition strategy during exercise) and opportunity (i.e., availability of aid stations, athlete support, etc.) which play a role in achieving nutrition targets. In multi-stage cycling events, it has been observed that athletes are successful in achieving aggressive fueling during competitions with the help of a support crew and regular nutrition provisions [19–21].

Endurance and ultra-endurance athletes experience a wide array of Ex-GIS with incidence ranging from 4 to 96% [22]. While numerous etiological and pathophysiological factors may contribute towards Ex-GIS, the redistribution of blood flow to skeletal muscle and extremities, and changes in the gastrointestinal nervous control, are considered primary mechanisms [17, 22]. Subsequent effects include intestinal epithelial damage and hyperpermeability, whole bacterial and bacterial endotoxin translocation, local and systemic inflammation, variations in gastrointestinal motility, and digestive/absorptive capacity changes, all of which have been characterized as part of the EIGS [17]. External factors such as environment conditions and exercise load (e.g., duration, intensity, and mode) may also increase the severity of EIGS and subsequent Ex-GIS [23–26]. Intrinsically, feeding at a period when gastrointestinal status is compromised may further exacerbate Ex-GIS. It has, however, been observed that elite athletes accustomed to feeding during exercise are able to tolerate greater intakes of food and fluid during exercise as compared with recreational athletes, and/or those who are not used to ingesting food during exercise [5, 27, 28]. As such, it is plausible that repetitive food and fluid intake during exercise that challenges the gastrointestinal tract during a period of compromised activity may lead to improvements in feeding tolerance, gastrointestinal comfort, Ex-GIS, and consequently better exercise performance outcomes.

**Fig. 1:**
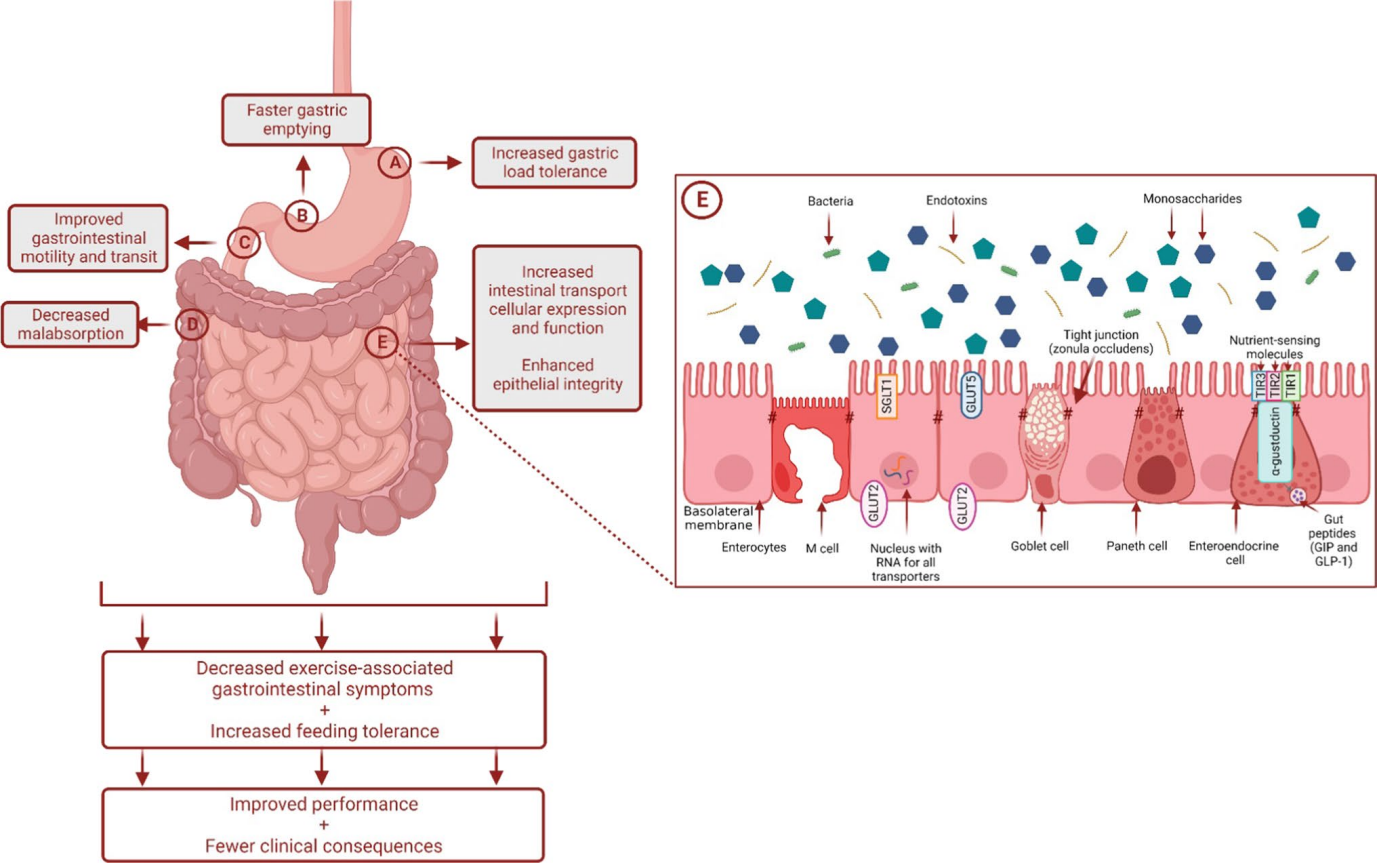
Schematic illustration of the potential mechanisms by which ‘gut-training’ or repetitive ‘feeding-challenge’ may provide beneficial outcomes in gastrointestinal integrity, function, systemic responses, and exercise-associated gastrointestinal symptoms (Ex-GIS). *SGLT1* sodium-glucose co-transporter 1, *GLUT5* glucose transporter 5, *GLUT2* glucose transporter 2, *TIR1* taste receptor type 1 member 1, *TIR2* taste receptor type 1 member 2, *TIR3* taste receptor type 1 member 3, *M cell* microfold cell, *GIP* gastric inhibitory peptide, *GLP* glucagon-like peptide-1

The concept of “*the gut being trained to cope with exercise*” previously proposed by Rehrer et al. in the 1990s [29], and followed up in the 2000s [30–32], suggests that the gastrointestinal system has the ability to adapt and improve its capacity to handle feedings during exercise through repetitive exposure to race-day nutrition conditions (Fig. 1). This notion stems from evidence that has shown nutrient-specific adaptations related to intestinal absorption in animal models [33–35], and enhancement of gastric emptying in non-athletic human populations [36, 37]. As an example, the increased messenger ribonucleic acid (mRNA) expression and protein synthesis of sodium-glucose co-transporter 1 (SGLT1), which is an important carbohydrate transporter predominantly found in the small intestine, has been shown in animal models to be mediated by constant exposure of the lumen to sugars, alongside the stimulation of intestinal nutrient sensing molecules (e.g., type 1 taste G protein-coupled receptors [GPCRs], specifically taste receptor type 1 member 3 [TIR3] and α-gustductin) [33, 34]. Similarly, in mice, glucose supplementation increased transport activity and mRNA levels of SGLT1 and glucose transporter 2 (GLUT2) [35]. Interestingly, specific sugar analogs have also been shown to influence certain intestinal glucose transport mRNA levels (i.e., methylglucose, d-galactose, d-mannose, and d-xylose on SGLT1; d-galactose and d-fructose on GLUT2) [35]. Using scintigraphy, gastric emptying of glucose was improved with short-term high-carbohydrate intake (3–7 days) among healthy adults [36, 37]. This can be attributed to inhibition of the negative feedback loop on gastric emptying and motility linked to a reduction in the sensitivity of nutrient sensing receptors or intestinal exposure to unabsorbed nutrients [38–40]. It is then plausible that if the gastrointestinal tract is repeatedly challenged during exercise with food, then intestinal absorptive capacity may be improved, and subsequently influence gastric emptying, thereby minimizing Ex-GIS. Reduction of Ex-GIS would lead to improved tolerance of nutritional intake that would enhance systemic and/or muscular fuel and could translate to improvements in performance outcomes [6]. As an example, greater oxidation of ingested carbohydrate was observed in a group of elite cyclists after training while eating a higher-carbohydrate diet for 28 days compared with a moderate carbohydrate intake [41]. While this study did not investigate gastrointestinal-related outcomes, it highlights that repeated exposure to high carbohydrate intake during training can impact physiological outcomes; specifically, substrate utilization. In reality, this form of targeted nutritional training is not entirely new for athletes, as practicing race-day nutrition during training is commonly given advice. However, gut-training entails doing this in a more structured and repetitive manner and is proposed to be a preventative and management strategy for EIGS and aligned Ex-GIS [30]. Previous narrative review publications on gut-training exist [30–32], which are generally speculative in nature and propose potential adaptation mechanisms following this type of targeted nutrition training. The supporting data presented in these reviews are also primarily based on animal experimental models due to limited human-focused research at that time. The method involves (i) training the stomach to hold larger volumes and improve its ability to cope with increased intra-gastric pressure, and subsequently, reduce the feeling of fullness and incidence or severity of upper gastrointestinal symptoms (e.g., gastric bloating, belching, upper abdominal pain, urge to regurgitate, regurgitation); (ii) facilitating a faster gastric emptying rate (GER) due to an increase in intra-gastric pressure tolerance and greater volume/concentration capture; and (iii) enhancing intestinal carbohydrate absorption through the increase in SGLT1 transcription and translation via gut hormone-regulating pathways involving gastric inhibitory polypeptide (GIP) and glucagon-like peptide-1 (GLP-1) linked to the stimulation of nutrient-sensing molecules in the intestine (e.g., T1R3 and α-gustductin) [34, 42]. All of these have implications on nutrient delivery to the muscles and subsequent use and Ex-GIS. While these adaptations seem promising, some of them have only been observed anecdotally (e.g., eating competitions) or as a result of a nutrition supplementation intervention without the exercise stress component [36, 37]. It is well known that exercise compromises gastrointestinal function and integrity [17]; thus, it is important to verify if these adaptations occur within an exercise model. Although gut-training has been a buzzword among sports practitioners and athletes in recent years, clear guidelines on how to implement this strategy before and during exercise are yet to exist.

Overall, the current literature is limited and lacks a complete and systematic review of gut-training (e.g., structured intervention) or feeding-challenge (e.g., acute intake) interventions that have been employed. In addition, the effect of gut-training or feeding-challenge on markers of gastrointestinal integrity and function, systemic immune response, and/or Ex-GIS has yet to be systematically assessed. Thus, this systematic literature review (SLR) aims to identify and synthesize research studies that have implemented structured and repetitive gut-training protocols or feeding-challenge/s before and/or during exercise in comparison with no feeding or feeding of lower volume or nutrient density, and its effect on markers of gastrointestinal integrity and/or function, systemic responses, and/or gastrointestinal symptoms.

## Methods

This systematic review was carried out in accordance with the Preferred Reporting Items for Systematic Reviews and Meta-Analyses (PRISMA) Statement [43].

### Search Strategy

A structured search strategy was developed with the assistance of an academic librarian and carried out across the following electronic databases from inception until May 2022: Ovid MEDLINE, EMBASE, CINAHL Plus, Web of Science Core Collection, and SPORTDiscus. Citation searching was also conducted among the identified studies to include any additional relevant studies. Additional information on the keywords used in the literature search for Ovid MEDLINE and EMBASE are given in Supplementary Information 1 (see electronic supplementary material [ESM]). Database search translation was done by adapting the search strategy to the unique features or functions of the other databases (e.g., CINAHL Plus, SPORTDiscus, and Web of Science Core Collection).

### Eligibility Criteria

To determine which studies were eligible for inclusion, the Participant Intervention Comparator Outcomes Study (PICOS) design format was used (Table 1). This review aimed to investigate healthy elite and recreationally competitive adolescent and adult (12–60 years) endurance athletes or athletes with running or cycling incorporated in their training. The intervention for inclusion entailed supplementing with carbohydrate food, with or without other nutrients, during exercise in a structured and repetitive manner (e.g., at least 2 consecutive days or across repeated trials) or feeding challenges conducted within 48 h before exercise (e.g., nutrition provision around exercise). The comparator was a control group consuming a placebo or carbohydrate in varying volume, texture, or nutritional density. Interventions that were excluded were those with dietary interventions involving high carbohydrate intake but not delivered in a structured and repetitive manner around exercise. In addition, outcomes of interest for inclusion were markers of systemic immune response, microbiota and gastrointestinal function, integrity, and symptoms. Only laboratory-controlled and field-based studies were considered, given the methodological details required, and those with incomplete data on the intervention (e.g., dose, frequency, duration) were excluded.

**Table 1:** Participant Intervention Comparator Outcomes Study (PICOS) criteria for study eligibility

PICOS	Inclusion	Exclusion
Population	Human Male and female biological sex Healthy individuals engaging in a structured physical activity or exercise program with an endurance component (> 90-min session) Recreational and competitive active adolescents and adults (aged 12–60 y)	Animal model and in vitro studies Infants Elderly Pregnant or lactating Sedentary individuals Clinical population (e.g., with disease or syndrome diagnosis) Individuals adhering to dietary modifications or using dietary supplements that would alter carbohydrate metabolism and gut microbiota (e.g., ketogenic diet, amylase inhibitors, pre-/pro-/syn-biotics)
Intervention	Structured and repetitive feeding of carbohydrates or ingestion of carbohydrate-containing fluids with or without the inclusion of other nutrients during exercise (e.g., for at least 2 consecutive days or across repeated trials) or food challenges conducted within 48 h before exercise (e.g., nutrition provision around exercise)	Dietary interventions involving high carbohydrate intake but not including structured and repetitive feeding of carbohydrate during exercise for at least 2 consecutive days or food challenges conducted within 48 h
Comparator	Placebo or control group or varying volume, texture, and nutritional density of supplement	
Outcome	*Gastrointestinal integrity markers* Damage/injury: I-FABP Permeability: claudin-3, zonulin, dual sugar tests – sucrose, lactulose & rhamnose/mannitol for small intestine, sucralose & erythritol for large intestine	
	*Gastrointestinal function markers* Emptying/motility: gastric aspiration, gastric myoelectrical signal via EGG, ^13^C acetate breath test, radio-isotope scanning OCTT: lactulose breath test Malabsorption (H_2_ & CH_4_ breath test) Transport activity: D-xylose and 3MG *Systemic immune response markers* Endotoxemia: LPS, LBP, sCD14, and/or EndoCAb Systemic inflammatory responses: plasma cytokine concentration *Microbiota* Bacterial taxonomy: ASV or OTU for relative abundance, bacterial diversity, microbial composition Functional markers/by-products: SCFA concentration (i.e. acetate, butyrate, and propionate) in plasma and feces *Gastrointestinal symptoms* Comfort and tolerance variables Upper, lower, and total symptoms	
Study design	Laboratory—controlled (RCT, comparative, parallel, or cross-over design) and field-based studies	All other study designs

*3MG* 3-O-methyl-D-glucose, *ASV* amplicon sequencing variants, *CH*_*4*_ methane, *EGG* electrogastrogram, *H*_*2*_ hydrogen, *I- FABP* intestinal-fatty acid binding protein, *LBP* lipopolysaccharide binding protein, *LPS* lipopolysaccharide, *OCTT* orocecal transit time, *OTU* operational taxonomic units, *RCT* randomized controlled trial, *sCD14* soluble CD14, *SCFA* short-chain fatty acid

### Study Selection

Search results from the different databases were imported into Endnote and any duplicates were removed. These were then imported into Covidence, an online systematic review software [44], to manage screening of studies by two reviewers (IM and AM), who worked independently and in duplicate to assess eligibility of identified papers against the PICOS tool. Any conflicts identified were resolved by a third reviewer (JB). Full-text papers for the screened studies were retrieved and evaluated against the PICOS model independently and in duplicate by two reviewers (IM and AM). Any disagreements were resolved through discussion with the third reviewer (JB).

### Data Extraction and Synthesis

Data from the included studies were independently extracted into a formatted table and cross-checked by two reviewers (IM and AM). Extracted data included general information about the study, study design and characteristics, nutrition- and exercise-related details of the intervention and comparisons, and primary and secondary outcomes of interest. Given the high level of heterogeneity of the study designs, nutrition and exercise interventions, measures and reported outcomes, data were synthesized and analyzed descriptively, and further analysis (e.g., meta-analysis) was not performed.

### Risk of Bias Assessment

Quality assessment of included studies was conducted using the Cochrane Collaboration’s risk-of-bias (RoB 2) tool [45]. This was performed independently and in duplicate by two reviewers (IM and AM) by referring to the criteria for judging risk of bias.

## Results

### Search Results

**Fig. 2:**
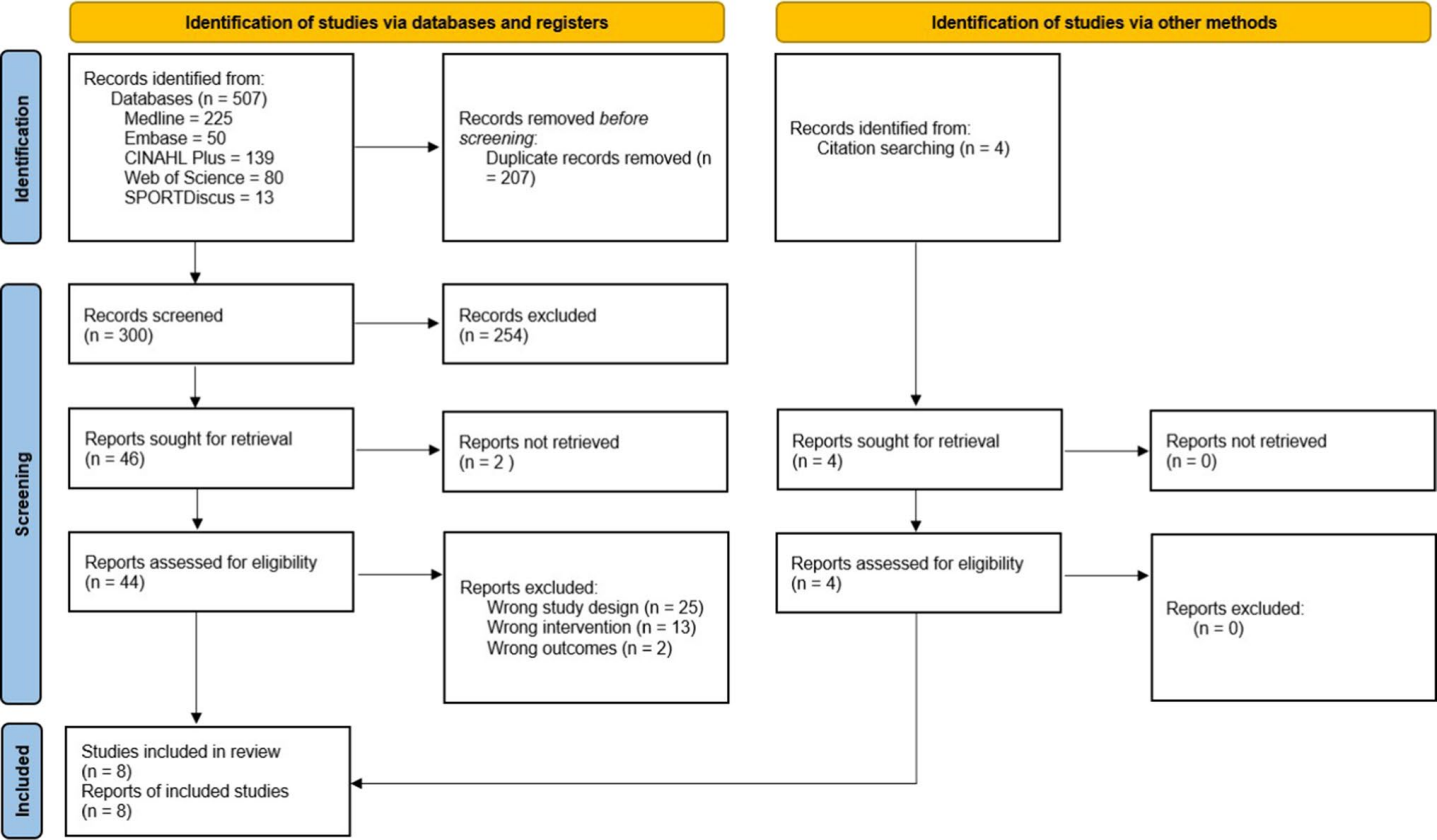
PRISMA diagram illustrating the systematic review process and the inclusion and exclusion of research papers

A total of 304 non-duplicate studies including those identified via citation searching were screened. After title and abstract screening, 254 were excluded. Only 48 of 50 studies sought for retrieval had a full-text version available and were then reviewed for eligibility. Studies were excluded due to wrong study design (*n* = 25), wrong intervention (*n* = 13) and wrong outcomes (*n* = 2). Overall, eight studies were included for review (Fig. 2).

### Study Characteristics

Participants in the included studies were between the ages of 15 and 35 years and were mostly males (79%). Almost all the studies were among cyclists, runners or triathletes with the exception of one which investigated elite junior Australian Rules Football players [46] and another which evaluated race walkers [28]. Exercise modalities used during gut-training or feeding-challenges included running (*n* = 5), cycling (*n* = 1), ball-skills sessions and intermittent running (*n* = 1), race walking (*n* = 1) or both running and cycling (*n* = 1), while one study among cyclists and triathletes did not disclose details on daily training sessions. Carbohydrate, specifically glucose only (*n* = 2), with electrolytes (*n* = 2), or with fructose (*n* = 3), was provided during exercise in varying amounts of 30–90 g/h (*n* = 7) or immediately before or during exercise at 1.5 g/kg/h (*n* = 1) in different forms (e.g., gels, solid food, and solutions). This was compared with either a carbohydrate-free consistency-matched placebo (*n* = 2), water (*n* = 1), a lower dose of carbohydrate (*n* = 4), carbohydrate with protein (*n* = 1), or the same amount of carbohydrate over repeated trials (*n* = 1). Additional details on study designs, intensity and duration of exercise training, and gut-training or feeding-challenge nutrition intervention are summarized in Table 2.

**Table 2:** General characteristics of included studies and gut-training or feeding-challenge protocol

Reference	Population	Study design	Intervention duration	Exercise load	Groups (gut-training/feeding-challenge vs comparator)
Costa et al. [5]	*n* = 25 recreationally competitive male and female runners	Randomized, placebo-controlled	2 weeks (5 training days followed by 2 rest days)	1-h running at 60% $\dot{V}{\rm O}_{2\max }$	CHO-S: 30-g gel (90 g/h) CHO-F: 30-g food portion equivalent (90 g/h) PLA: matched formulated gel consumed every 20 min with water (10% *w*/*v*)
Cox et al. [50]	*n* = 16 well trained male cyclists and triathletes	Randomized, controlled	28 days	Daily training details not disclosed	Higher CHO: 1.5 g CHO/kg/h immediately before or during exercise Moderate CHO: fasted 2 h before exercise and no carbohydrate during exercise
Houmard et al. [47]	*n* = 10 well trained male biathletes	Crossover	4 repeated trials	1-h running at 75% $\dot{V}{\text{O}}_{2\max }$ or 1-h cycling at 75% $\dot{V}{\text{O}}_{2\max }$	RC: 7% CHO solution, 180 mL/15 min (51.7 g/h) while running CYC: 7% CHO solution, 180 mL/15 min (51.7 g/h) while cycling RW: 180 mL/15 min of water while running CYW: 180 mL/15 min of water while cycling
King et al. [28] study 1	*n* = 19 elite male race walkers	Randomized, controlled	14 days	Two 25-km + walks, 10 × 1000-m efforts on a 6-min cycle, and a tempo hill session	CON: 8% CHO solution (30 g/h) MAX: 24% CHO solution (60–90 g/h)
King et al. [28] study 2	*n* = 18 elite male and female runners	Randomized, controlled	14 days	Twice-weekly long runs, high intensity running, recovery runs, and gym sessions	CON: 30 g/h CHO solution MAX: 60–90 g/h hydrogel CHO
Lambert et al. [48]	*n* = 7 trained male and female runners	Repeated trials	6 trials	90-min running at 65% $\dot{V}O_{2\max }$	Run 1: Ad libitum drinking Runs 2–6: 4% CHO-electrolyte solution ingested every 10 min ~ 178 mL/10 min (57 g/90 min)
Lee et al. [46]	*n* = 21 high-level junior male Australian Rules Football players	Double-blind, placebo-controlled, randomized	2 training days and 1 match day	60-min low-intensity ball-skills session (training) 90-min intermittent exercise (match)	CHO: 1.2 g/kg/h CHO during training (beverage) and matches (beverage and solid feedings) CHO + PRO: 0.87 g/kg/h CHO + 0.33 g/kg/h PRO during training (beverage) and matches (beverage and solid feedings)
Miall et al. [49]	*n* = 18 recreationally competitive male runners	Randomized, placebo-controlled	2 weeks (5 training days followed by 2 rest days)	1-h running at 60% $\dot{V}O_{2\max }$	CHO: 30-g gel (90 g/h) PLA: 0 matched formulated gel consumed every 20 min with water (10% w/v)
Svendsen et al. [51]	*n* = 13 highly trained male cyclists	Double-blind, randomized, crossover	8 days	Two sessions per day, high-intensity cycling (Zones 3–5, 82 to > 92% HR_max_) for ~ 35% of session	H-CHO: 118 mL of 20% CHO solution (24 g) before exercise and 1 L/h of 6% CHO solution (60 g/h) during exercise L-CHO: 118 mL of 2% CHO solution (2.4 g) before, and 1 L/h of 2% CHO solution (20 g/h) during exercise

*CHO* carbohydrate (group), *CHO-F* carbohydrate-food group, *CHO* + *PRO* carbohydrate and protein group, *CHO-S* carbohydrate-supplement group, *CON* control group, *CYC* cycling with carbohydrate trial, *CYW* cycling with water trial, *H-CHO* high carbohydrate group, *HR*_*max*_ maximum heart rate, *L-CHO* low carbohydrate group, *MAX* maximum carbohydrate group, *PLA* placebo group, *PRO* protein, *RC* running with carbohydrate trial, *RW* running with water trial, $\dot{V}O_{2\max }$ maximal oxygen uptake

### Effect of Gut-Training or Feeding-Challenges on Gastrointestinal Integrity

Three studies assessed the effect of gut-training or feeding-challenges on markers of gastrointestinal integrity in response to exercise (Table 3). All three studies measured plasma intestinal-fatty acid binding protein (I-FABP) concentration, while one included plasma claudin-3 as well. Results from the study by Costa et al. [5], wherein runners underwent two weeks of gut-training, were inconclusive. In this study, plasma I-FABP concentration in response to 3 h of exercise decreased by 13% (pre: 981 pg/mL, post: 848 pg/mL) and 16% (pre:1243 pg/mL, post: 1040 pg/mL) in the carbohydrate-supplement and placebo groups, respectively, and increased by 18% (pre: 1236 pg/mL, post: 1462 pg/mL) in the carbohydrate-food group post-intervention. These were, however, not significantly different over time and between groups. The first study by King et al. [28] among race walkers failed to demonstrate a significant change in plasma I-FABP concentration in response to exercise between trials or groups. In for the second study by King et al. [28] on runners, a 44% increase in the control group and a 4% decrease in the maximum carbohydrate group in plasma I-FABP concentrations were observed but was not significantly different between groups. Furthermore, plasma claudin-3 response to 28 km of steady-state running followed by a 7-km self-paced time trial similarly decreased post-intervention in both groups. Overall, the exercise stress and nutrition used were highly variable across the studies.

**Table 3:** Effect of gut-training or feeding-challenges on gastrointestinal integrity in response to exercise

Reference	Population	Exercise protocol	Provision of meal and (or) fluid	Findings
Costa et al. [5]	*n* = 25 recreationally competitive male and female runners	2-h steady-state running (60% $\dot{V}{\text{O}}_{2\max }$) + 1-h self-paced distance test	30 g of CHO-S or CHO-F or PLA every 20 min (90 g/h) + water (10% *w*/*v*) Water ad libitum during distance test	Ø I-FABP
King et al. [28] study 1	*n* = 19 elite male race walkers	26-km race walking	Pre-intervention: 8% CS pre-exercise and during exercise (30 g/h for CON and MAX) Post-intervention:: 8% and 24% CS pre-exercise and during exercise (30 g/h for CON and 90 g/h for MAX)	Ø I-FABP
King et al. [28] study 2	*n* = 18 elite male and female runners	28-km steady-state running (80% of predicted marathon speed) + 7-km self-paced time trial	Pre-intervention: HCS every 3.5 km (30 g/h for CON and MAX) Post-intervention: HCS every 3.5 km (30 g/h for CON and 70–100 g/h for MAX)	Ø I-FABP Ø Claudin-3

*CHO-F* carbohydrate-food group, *CHO-S* carbohydrate-supplement group, *CON* control group, *CS* carbohydrate solution, *HCS* hydrogel carbohydrate solution, *I-FABP* intestinal fatty acid binding protein, *MAX* maximum carbohydrate group, *PLA* placebo group, $\dot{V}O_{2\max }$ maximal oxygen uptake, Ø indicates no significant difference between trials and groups

### Effect of Gut-Training or Feeding-Challenges on Gastrointestinal Functional Responses

Two studies measured GER in repeated or crossover trials, while four studies measured carbohydrate malabsorption using breath hydrogen (H_2_) concentration pre- and post-intervention (Table 4). In the study by Houmard et al. [47], GER of both water and 7% carbohydrate solution did not differ significantly between the running or cycling trials at 75% maximal oxygen uptake ($\dot{V}O_{2\max }$), as well as over time. Lambert et al. [48] also showed no significant difference in GER of a 4% carbohydrate-electrolyte solution across the repeated trials among runners (12.1 ± 1.9 mL/min in trial 2 versus 12.3 ± 2.3 mL/min in trial 6). In terms of carbohydrate malabsorption, improvements were observed post-intervention in two studies. In the study by Costa et al. [5], breath H_2_ peak response to exercise was significantly lower by 45% after 2 weeks of gut-training only in the carbohydrate-supplement group but not in the carbohydrate-food and placebo groups. Similarly, Miall et al. [49] also showed a significant decrease of 54% in breath H_2_ peak in the carbohydrate group but not the placebo group after 2 weeks of gut-training. In both the Costa et al. [5] and Miall et al. [49] studies, the post-intervention peak breath H_2_ values in the carbohydrate-supplement group and carbohydrate group, respectively, were < 10 ppm, which is considered clinically insignificant in terms of carbohydrate malabsorption. Lastly, both studies conducted by the King et al. group [28] collected breath samples for H_2_ concentration; however, only results from the first study among race walkers were reported. In both the control and the carbohydrate maximum groups, there were no significant changes in breath H_2_ during the 26-km race walking protocol and the post-exercise period after the intervention. The number of participants with breath H_2_ concentrations above clinical threshold for carbohydrate malabsorption (> 10 ppm above basal reading on two consecutive occasions) in the control group was 3 out of 10 at pre- and post-intervention, while in the carbohydrate maximum group, there were 5 out of 9 at pre-intervention and 4 out of 9 at post-intervention.

**Table 4:** Effect of gut-training or feeding-challenges on gastrointestinal functional responses in response to exercise

Reference	Population	Exercise protocol	Provision of meal and (or) fluid	Findings
Gastric emptying				
Houmard et al. [47]	*n* = 10 well trained, male biathletes	1-h running or cycling (75% $\dot{V}O_{2\max }$)	7% CS, 180 mL/15 min (51.7 g/h) or 180 mL water/15 min	Ø GER
Lambert et al. [48]	*n* = 7 trained male and female runners	90-min running (65% $\dot{V}O_{2\max }$^)^	4% CES, 1246 mL/90 min (33 g/h)	Ø GER
Carbohydrate malabsorption				
Costa et al. [5]	*n* = 25 recreationally competitive male and female runners	2-h steady-state running (60% $\dot{V}{\text{O}}_{2\max }$) + 1-h self-paced distance test	30 g of CHO-S or CHO-F or PLA every 20 min (90 g/h) + water (10% *w*/*v*) Water ad libitum during distance test	↓ H_2_ peak post-intervention in CHO-S
King et al. [28] study 1	*n* = 19 elite male race walkers	26-km race walking	Pre-intervention: 8% CS pre-exercise and during exercise (30 g/h for CON and MAX) Post-intervention: 8% and 24% CS pre-exercise and during exercise (30 g/h for CON and 90 g/h for MAX)	Ø Breath H_2_
King et al. [28] study 2	*n* = 18 elite male and female runners	28-km steady-state running + 7-km self-paced time trial	Pre-intervention: HCS every 3.5 km (30 g/h for CON and MAX) Post-intervention: HCS every 3.5 km (30 g/h for CON and 70–100 g/h for MAX)	*No results reported*
Miall et al. [49]	*n* = 18 recreationally competitive male runners	2-h steady-state running (60% $\dot{V}{\text{O}}_{2\max }$) + 1-h self-paced distance test	30 g of CHO-S (90 g/h) or PLA every 20 min + water 10% *w*/*v* Water ad libitum during distance test	↓ H_2_ peak post-intervention in CHO-S

*CES* carbohydrate electrolyte solution, *CHO* carbohydrate group, *CHO-S* carbohydrate gel-disc, *CHO-F* carbohydrate-food, *CON* control group, *CS* carbohydrate solution, *GER* gastric emptying rate, *H*_*2*_ hydrogen, *HCS* hydrogel carbohydrate solution, *MAX* maximum carbohydrate group, *PLA* placebo, $\dot{V}O_{2\max }$ maximal oxygen uptake; **↓** indicates significant decrease with gut-training vs comparator, Ø indicates no significant difference between trials and groups

### Effect of Gut-Training and Feeding-Challenges on Systemic Immune Response

Two studies [50, 51] included measures of systemic immune responses (Table 5). After a 28-day intervention, results from the study by Cox et al. [50] showed mixed cytokine response to 100 min of cycling at 70% $\dot{V}O_{2\max }$ plus a 7-kJ/kg time trial. For cytokine response to exercise, interleukin (IL)-6 was lower in both the higher-carbohydrate and moderate-carbohydrate groups (17% and 27%, respectively), while IL-8 increased in both groups (7% and 3%, respectively) at post-intervention, but these were not statistically significant. Anti-inflammatory cytokine response, namely, IL-10 and IL-1 receptor antagonists, was reduced in both the moderate-carbohydrate group (31% and 47%, respectively) and higher-carbohydrate group (6% and 13%, respectively) at post-intervention; however, these were not statistically significant. In the study by Svendsen et al. [51], it is important to note that systemic immune response was measured at rest in response to antigen challenge following 8 days of intensified training with either a low-carbohydrate or high-carbohydrate intake. At post-intensified training, high-carbohydrate condition compared with the low-carbohydrate condition had a significantly lower IL-1α (0.33 vs 0.70 pg/mL) and IL-1β (6.0 vs 9.3 pg/mL) production. Tumor necrosis factor alpha (TNFα) also increased more in the low-carbohydrate condition compared with the high-carbohydrate condition, but this was not statistically significant. Lastly, no significant differences were observed in IL-2, IL-4, IL-6, IL-8, and interferon gamma (IFN- γ) after intensified training in both conditions.

**Table 5:** Effect of gut-training or feeding-challenges on systemic immune response to exercise

Reference	Population	Exercise protocol	Provision of meal and (or) fluid	Findings
Cox et al. [50]	*n* = 16 well trained male cyclists and triathletes	100 min of cycling (70% $\dot{V}{\text{O}}_{2\max }$) + time trial at a pre-determined workload (7 kJ/kg)	15 mL/kg/h of water every 20 min (in 5 mL/kg volumes) + 5 mL/kg of water during time trial	IL-6: decreased ^ns^ IL-8: increase ^ns^ IL-10: decreased in moderate-CHO group, increased post-exercise and decreased 1-h post-exercise in higher-CHO group ^ns^ IL-1ra: decreased in moderate-CHO group, increased post-exercise and decreased 1-h post-exercise in higher-CHO group ^ns^
Svendsen et al. [51]	*n* = 13 highly trained male cyclists	At rest following intensified training	N/A	↑ IL-1α (L-CHO > H-CHO) ↑ IL-1β (L-CHO > H-CHO) TNFα: increased (L-CHO > H-CHO) ^ns^ Ø IL-2, IL-4, IL-6, IL-8, and IFN-γ

*H-CHO* high carbohydrate, *IFN-γ* interferon gamma, *IL* interleukin, *IL-1ra* interleukin-1 receptor antagonist, *L-CHO* low carbohydrate, *TNFα* tumor necrosis factor alpha, $\dot{V}O_{2\max }$ maximal oxygen uptake; ↑ indicates significant increase with gut-training vs comparator, ^ns^ indicates no significant difference between gut-training and comparator, Ø indicates no significant difference between trials and groups

### Effect of Gut-Training or Feeding-Challenges on Gastrointestinal Symptoms and Feeding Tolerance

Five studies had Ex-GIS as outcomes, which were measured using varied methods (Table 6) before, during, and/or after exercise. A significant reduction in gut discomfort during exercise post-intervention was seen in both the carbohydrate-supplement (44%) and carbohydrate-food (49%) groups during steady-state running, distance test, and recovery in the Costa et al. [5] study, and in the carbohydrate group (48%) in the Miall et al. [49] study compared with placebo (18% and 20%, respectively). Gut discomfort during exercise was also significantly reduced by 26% after repeated trials in the study by Lambert et al. [48]. Lee et al. [46] showed that the median score of post-match gut discomfort did not significantly differ across all football matches between players who ingested carbohydrate only and those who ingested carbohydrate and protein. Total gastrointestinal symptoms during and/or after exercise were significantly reduced post-intervention in the carbohydrate-supplement (60%) and carbohydrate-food (63%) groups in the study by Costa et al. [5] and in the carbohydrate group (61%) in the Miall et al. [49] study compared with placebo (25% and 2%, respectively). In the first study by King et al. [28] among race walkers, total gastrointestinal symptoms were significantly greater post-intervention versus pre-intervention at 13 km, 19 km, and post-exercise in the maximum carbohydrate group. In the control group, no significant differences were observed between trials and time points. The second study by King et al. [28] among runners showed that total gastrointestinal symptoms were similarly increased across exercise in both trials and groups. Lee et al. [46] used a 4-point Likert-type scale and reported that severe symptoms were not present in any of the groups or matches with no numerical data provided. Regarding upper gastrointestinal symptoms during and/or after exercise, these were significantly reduced post-intervention in the carbohydrate-supplement (64%) and carbohydrate-food (62%) groups in the Costa et al. [5] study and in the carbohydrate group (70%) in Miall et al. [49] study compared with placebo (25% and 10%, respectively). In the study by King et al. [28], upper gastrointestinal symptoms among the race walkers during and/or after exercise were significantly higher post-intervention compared with pre-intervention in the maximum carbohydrate group, but among the runners, this was significantly greater in the control group versus the maximum carbohydrate group in both trials. Costa et al. [5] demonstrated a significant decrease in lower gastrointestinal symptoms during and/or after exercise post-intervention in the carbohydrate-supplement (40%) and carbohydrate-food (70%) groups compared with placebo (60%) using a 10-point Likert-type scale. In the study by Miall et al. [49], where they used a 10-point Likert-type scale, lower gastrointestinal symptoms during and/or after exercise were significantly reduced in the carbohydrate group (39%) and increased in the placebo group (50%). King et al. [28] used a modified visual analog scale (mVAS) and showed that among the race walkers, lower gastrointestinal symptoms during and/or after exercise were significantly decreased post-intervention in the maximum carbohydrate group, while no significant changes were observed in the control group. In the study on runners by King et al. [28], both groups had greater lower gastrointestinal symptoms during and/or after exercise post-intervention compared with pre-intervention. Other gastrointestinal symptoms reported were a significant reduction in nausea during and/or after exercise in the carbohydrate-supplement (79%) and carbohydrate-food (59%) groups compared with placebo in the Costa et al. [5] study. In the Lambert et al. [48] study, wherein they used a 133-mm (mm) scale, symptoms of nausea (1 ± 3 mm), heartburn (0 ± 3 mm) and abdominal cramps (30 ± 16 mm) were noted during exercise, but these were not significantly different across trials. Lastly, feeding tolerance variables during and/or after exercise were similar between pre- and post-intervention in both groups in the Miall et al. [49] study, while thirst and fullness were also similar across trials in the Lambert et al. [48] study.

**Table 6:** Effect of gut-training or feeding-challenges on gastrointestinal symptoms in response to exercise

Reference	Population	Method	Gut discomfort	Total GIS	Upper GIS	Lower GIS	Other GIS	Feeding tolerance
Costa et al. [5]	*n* = 25 recreationally competitive male and female runners	10-point Likert-type scale	↓ Post-intervention in CHO-S and CHO-F vs PLA	↓ Post-intervention in CHO-S and CHO-F vs PLA	↓ Post-intervention in CHO-S vs PLA	↓ Post-intervention in CHO-S vs PLA	↓ Nausea post-intervention in CHO-S vs PLA (100% increase)	
King et al. [28] study 1	*n* = 19 elite male race walkers	mVAS 10-point Likert-type scale		↑ Post-intervention in MAX vs CON MAX: Post-intervention > pre-intervention at 13 and 19 km and post-exercise Ø CON	↑ Post-intervention in MAX vs CON MAX: Post-intervention > pre-intervention at 13 and 19 km and post-exercise Ø CON	↑ Post-intervention in MAX vs CON MAX: Post-intervention > pre-intervention Ø CON		
King et al. [28] study 2	*n* = 18 elite male and female runners	*Same as above*		Increased across exercise in both CON and MAX at pre- and post-intervention ^ns^	Increased across exercise, CON > MAX pre- and post-intervention	Increased post-intervention in both CON and MAX vs pre-intervention ^ns^		
Lambert et al. [48]	*n* = 7 trained male and female runners	VAS (133 mm)	↓ Gut discomfort				Ø Nausea, heartburn,urge to defecate, and abdominal cramps	Ø Thirst and fullness
Lee et al. [46]	*n* = 21 high-level, junior male Australian Rules Football players	4-point Likert-type scale	Ø Gut discomfort	No report of severe symptoms				
Miall et al. [49]	*n* = 18 recreationally competitive male runners	10-point Likert-type scale	↓ Post-intervention in CHO vs PLA	↓ Post-intervention in CHO vs PLA	↓ Post-intervention in CHO vs PLA	↓ Post-intervention in CHO vs PLA		Ø Thirst, fullness, tolerance to food/drink

*CHO* carbohydrate group, *CHO-F* carbohydrate-food group, *CHO-S* carbohydrate-supplement group, *CON* control group, *GIS* gastrointestinal symptoms, *MAX* maximum carbohydrate group, *mVAS* modified visual analog scale, *PLA* placebo group, *VAS* visual analog scale; ↑ indicates significant increase with gut-training vs comparator, **↓** indicates significant decrease with gut-training vs comparator, Ø indicates no significant difference between trials and groups, ^ns^ indicates no significant difference between gut-training and comparator

### Effect of Gut-Training or Feeding-Challenges on Glucose Availability and/or Muscle Fuel Kinetics and Exercise Performance

Glucose availability was measured in three studies while only one study investigated muscle fuel kinetics (Table 7). In the study by Costa et al. [5], post-intervention blood glucose concentration after 2 h of steady-state running at 60% $\dot{V}O_{2\max }$ followed by a 1-h self-paced distance test was significantly higher in the carbohydrate-supplement group (7.2 mmol/L) compared with the carbohydrate-food (6.1 mmol/L) and placebo (6.2 mmol/L) groups. In addition, after the gut-training intervention, blood glucose concentration was also significantly higher in the carbohydrate-supplement group at the 90-min timepoint during the 2-h run compared with the placebo group (6.6 vs 5.9 mmol/L, respectively). In contrast, in the Cox et al. study [50], there were no significant differences between the moderate-carbohydrate group and higher-carbohydrate group in pre- (4.9 and 4.7 mmol/L, respectively) and post-exercise (4.7 and 4.5 mmol/L, respectively) plasma glucose concentration before the intervention. Likewise, post-intervention plasma glucose concentrations in the moderate-carbohydrate group and higher-carbohydrate group before (4.7 vs 4.6 mmol/L, respectively) and after exercise (4.6 vs 5.0 mmol/L, respectively) were not significantly different. The exercise protocol in this study was 100 min of cycling at 70% $\dot{V}{\rm O}_{2\max }$ followed by a time trial at a pre-determined workload of 7 kJ/kg. In the study by Houmard et al. [47], blood glucose level after 1 h of cycling at 75%$\dot{V}{\rm O}_{2\max }$ was significantly higher in the carbohydrate trial (5.6 mmol/L) compared with the water trial (4.4 mmol/L). No significant differences in post-exercise blood glucose levels were observed between the running with carbohydrate trial (5.2 mmol/L) and the running with water trial (4.7 mmol/L). Muscle fuel kinetics was only assessed in the study by Costa et al. [5], wherein they found no significant differences between groups in total fat and carbohydrate oxidation during 3-h running at pre- and post-intervention.

**Table 7:** Effect of gut-training or feeding-challenges on glucose availability and/or muscle fuel kinetics in response to exercise

Reference	Population	Exercise protocol	Provision of meal and (or) fluid	Findings
Blood glucose availability				
Costa et al. [5]	*n* = 25 recreationally competitive male and female runners	2 h of steady-state running (60% $\dot{V}{\text{O}}_{2\max }$) followed by a 1-h self-paced distance test	30 g of CHO-S or CHO-F or PLA every 20 min (90 g/h) + water (10% *w*/*v*) Water ad libitum during distance test	↑ Blood glucose post-intervention CHO-S at 90 min of steady state running vs PLA ↑ Blood glucose post-intervention in CHO-S after distance test vs CHO-F and PLA
Cox et al. [50]	*n* = 16 well trained male cyclists and triathletes	100 min of cycling (70% $\dot{V}{\rm O}_{2\max }$) + time trial at a pre-determined workload (7 kJ/kg)	15 mL/kg/h water every 20 min (in 5 mL/kg volumes) + 5 mL/kg of water during time trial	Ø Blood glucose
Muscle fuel kinetics				
Costa et al. [5]	*n* = 25 recreationally competitive male and female runners	2 h of steady-state running (60% $\dot{V}{\text{O}}_{2\max }$) followed by a 1-h self-paced distance test	30 g CHO-S or CHO-F or PLA every 20 min (90 g/h) + water (10% *w*/*v*) Water ad libitum during distance test	Ø Total carbohydrate and fat oxidation during steady-state running and distance test
Exercise performance				
Costa et al. [5]	*n* = 25 recreationally competitive male and female runners	2 h of steady-state running (60% $\dot{V}{\text{O}}_{2\max }$) followed by a 1-h self-paced distance test	30 g CHO-S or CHO-F or PLA every 20 min (90 g/h) + water (10% *w*/*v*) Water ad libitum during distance test	↑ Distance covered in 1-h distance test
Cox et al. [50]	*n* = 16 well trained male cyclists and triathletes	100 min of cycling (70% $\dot{V}{\rm O}_{2\max }$) + time trial at a pre-determined workload (7 kJ/kg)	15 mL/kg/h water every 20 min (in 5 mL/kg volumes) + 5 mL/kg of water during time trial	Ø Max time and PPO
King et al. [28] study 2	*n* = 18 elite male and female runners	28 km of steady-state running + 7-km self-paced time trial	Pre-intervention: HCS every 3.5 km (30 g/h for CON and MAX) Post-intervention: HCS every 3.5 km (30 g/h for CON and 70–100 g/h for MAX)	Ø Mean time
Lee et al. [46]	*n* = 21 high-level male junior Australian Rules Football players	1 set of 5 CMJ, with 1–3 s between each jump throughout the 7-day tournament	N/A	↑ Peak velocity in CHO + PRO Ø Mean power, mean force and jump height
Miall et al. [49]	*n* = 18 recreationally competitive male runners	2 h of steady-state running (60% $\dot{V}{\text{O}}_{2\max }$) + 1-h self-paced distance test	30 g of CHO (90 g/h) or PLA every 20 min + water 10% w/v Water ad libitum during distance test	↑ Distance covered in 1-h distance test
Svendsen et al. [51]	*n* = 13 highly trained male cyclists	Continuous, incremental test to volitional exhaustion (60 W with 35 W increments every 3 min)	N/A	Ø $\dot{V}{\text{O}}_{2\max }$ ↓ peak power post-IT

*CHO* carbohydrate group, *CHO-F* carbohydrate-food, *CHO* + *PRO* carbohydrate and protein group, *CHO-S* carbohydrate gel-disc, *CMJ* countermovement jumps, *CON* control group, *H-CHO* high-carbohydrate, *IT* intensified training, *L-CHO* low-carbohydrate, *MAX* maximum carbohydrate group, *Max time* time (min) to reach $\dot{V}{\text{O}}_{2\max }$, *PLA* placebo, *PPO* peak power output (watts), $\dot{V}O_{2\max }$ maximal oxygen uptake; ↑ indicates significant increase with gut-training vs comparator, Ø indicates no significant difference between trials and groups

Exercise performance outcomes were included in six studies (Table 7). Improvements in performance were observed in both the studies by Costa et al. [5] and Miall et al. [49], wherein the carbohydrate gut-training groups covered more distance in a 1-h self-paced running distance test after the intervention, compared with the placebo group. In contrast, mean time to complete a 7-km self-paced time trial remained unchanged after a similar 2-week gut-training protocol in the King et al. [28] study among runners. Cox et al. [50] observed similar improvements (~ 9%) in a cycling time trial task (7 kJ/kg) that followed 100 min of steady-state (70% $\dot{V}{\rm O}_{2\max }$) cycling in both the moderate-carbohydrate group and higher-carbohydrate group. In the study by Svendsen et al. [51] among highly trained cyclists, a continuous incremental test to volitional exhaustion starting at 60 W with 35-W increments every 3 min was performed before and after the 8-day intensified training period. No significant difference in $\dot{V}{\rm O}_{2\max }$ was observed between the high-carbohydrate and low-carbohydrate groups but a 4% decrease in peak power was observed after the 8-day intensified training in both conditions. Lastly, in the study by Lee et al. [46], neuromuscular performance was assessed throughout the 7-day tournament using linear position transducers to measure countermovement jumps. An increase in countermovement jump peak velocity was observed in the carbohydrate and protein group but not in the carbohydrate group, whereas jump height, mean force, and power remained similar throughout the tournament for both groups.

### Risk of Bias Assessment

Certain studies did not explicitly state their blinding and randomization procedures, or it was not possible to entirely blind participants or the researchers, and thus were assessed as having ‘some concerns’ in certain domains [45]. These included period and carryover effects, effects of adhering to intervention, and measurement of outcome, but most of the included studies had an overall low risk of bias (Table 8).

**Table 8:**
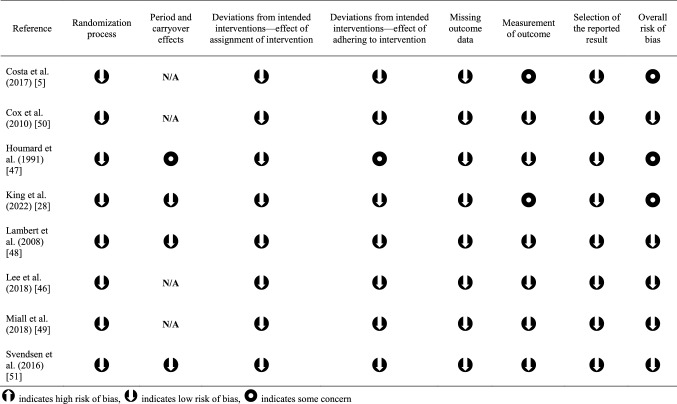
Risk of bias assessment results

## Discussion

### Overall Outcomes

**Fig. 3:**
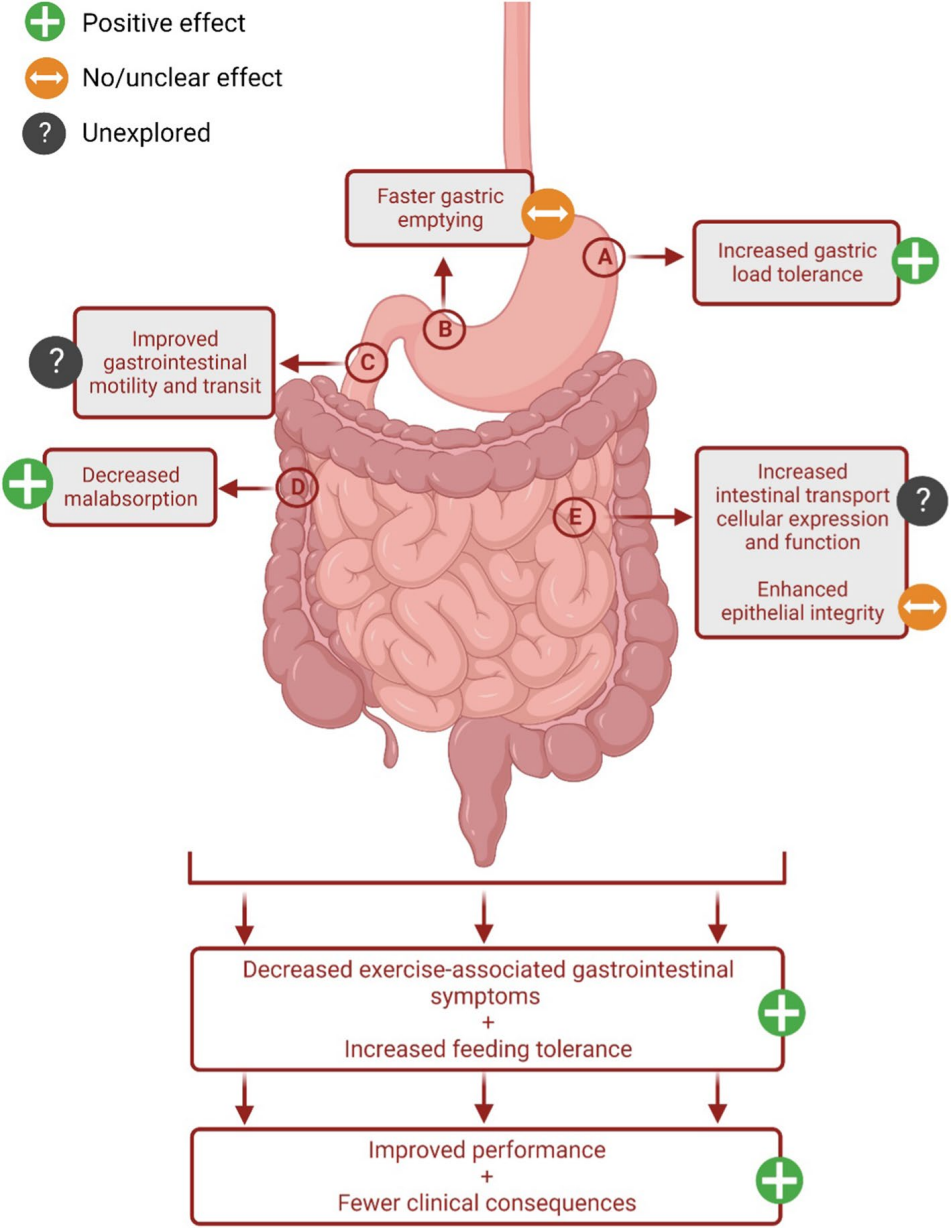
Schematic illustration of the systematic literature review outcome by which ‘gut-training’ or repetitive ‘feeding-challenge’ affects gastrointestinal integrity, function, systemic responses, and exercise-associated gastrointestinal symptoms (Ex-GIS)

The current SLR aimed to identify and synthesize research studies that have implemented structured and repetitive gut-training protocols or feeding-challenge/s around exercise and assessed their effect on markers of gastrointestinal integrity and/or function, systemic responses, and/or gastrointestinal symptoms. A total of 304 studies were identified and screened, out of which, eight were included in the review. Results from the current SLR showed that gut-training or feeding-challenge around exercise does not enhance gastrointestinal integrity based on the lack of significant changes in measures of plasma I-FABP and claudin-3 in the included studies. Furthermore, in terms of gastrointestinal function, it appears that gut training or feeding-challenge around exercise does not make GER faster, but reduces carbohydrate malabsorption, as evidenced by the reduction of peak breath H_2_ response to carbohydrate feeding during exercise. The effect of gut-training or feeding-challenge around exercise on systemic immune response remains unclear with studies having mixed results. Lastly, gut- training and feeding-challenge significantly improved gut discomfort during and/or after exercise, while only certain studies showed positive effects on Ex-GIS. There is also limited evidence to imply that gut-training or feeding-challenges around exercise positively impacts blood glucose availability and exercise performance. Overall, it seems that repetitively exposing the gastrointestinal tract to food and fluid during exercise, which challenges it during a period of compromised status, leads to positive functional and symptomatic outcomes that may translate to improved nutritional intake, which in theory, would have exercise performance implications (Fig. 3).

### Gastrointestinal Integrity

Exercise stress compromises gastrointestinal integrity [13, 15]. During exercise, blood flow is diverted away from the gastrointestinal system and redirected towards working muscles and the extremities, causing gastrointestinal hypoperfusion and ischemia [52, 53]. As a result, intestinal epithelial injury occurs and gastrointestinal permeability is increased, which is characteristic of EIGS [17]. Results from the current SLR show that gut-training or feeding-challenge interventions did not enhance gastrointestinal integrity based on measures of plasma I-FABP concentration in three studies and plasma claudin-3 in one study. Disturbances in gastrointestinal integrity are commonly measured using plasma/serum I-FABP for intestinal epithelial injury, fecal calprotectin for intestinal epithelial inflammation, and fecal/plasma claudin-3 for intestinal tight junction injury and/or dysfunction [54–56]. Alternative measures that could potentially be more sensitive or specific for intestinal permeability (e.g., non-metabolizable sugar probes like lactulose with L-rhamnose or mannitol for small intestine permeability) [52, 57], or endotoxemia-related surrogate markers of intestinal epithelial permeability such as circulating lipopolysaccharides (LPS; a component of the outer membrane of Gram-negative bacteria), lipopolysaccharide binding protein (LBP), soluble CD14 (sCD14), and endogenous endotoxin-core antibody (EndoCAb) [58–62], are yet to be explored. Interestingly, despite the reduction in small intestinal epithelial permeability with feeding during exercise that has previously been shown [9], this outcome was not observed in the studies included in this review. Changes in circulating concentrations of I-FABP are reflective of intestinal epithelial cell injury, as this protein is released when cells such as enterocytes are damaged and this has been shown to substantially increase with exertion and exertional-heat stress [63, 64]. The pre- to post-exercise increase in plasma I-FABP concentrations before and after a 2-week gut-training ranged from 82 to 4443 pg/mL [18], showing considerable individual variation. Some participants presented responses reflective of a protective effect (e.g., attenuated I-FABP response), likely attributed to the carbohydrate feeding during exercise [9], and other responses of clinical significance (Δ ≥ 1000 pg/mL) [65]. Considering the inverse association between epithelial injury with gut discomfort and Ex-GIS reported in this study, the authors suggested these outcomes may be due to individual tolerance to the transit and trafficking of gastrointestinal content as a result of the during-exercise feeding protocol. In participants with poor tolerance, it is plausible that greater intestinal nutrient content promotes a nutrient mediator for hyperemia in intestinal villi microvasculature, previously demonstrated when carbohydrate is provided frequently during exercise [9, 66, 67] and vice versa, as shown in highly trained endurance athletes accustomed to consuming high carbohydrate loads during exercise and showing good tolerance [27, 28].

Gastrointestinal permeability to pathogenic lumen bacterial endotoxins is related to intestinal epithelial cell structure stability, but also to intestinal epithelial tissue tight-junction protein stability and regulation, of which claudin-3 is used as a surrogate biomarker [68]. It is generally thought that increased plasma claudin-3 concentration is related to the magnitude of intestinal epithelial tissue hyperpermeability [56]. However, recent experimental evidence using a 3-h steady-state running protocol reported no change in plasma claudin-3 responses, despite substantial increases in biomarkers of intestinal injury, endotoxemia, and cytokinemia [69]. It is therefore not surprising that similar changes in claudin-3 levels post-intervention were observed in both groups in the King et al. [28] study. A large individual variation, likely associated with confounding aspects of the mechanism instigating epithelial injury and gastrointestinal permeability response, could possibly explain the insignificant findings on the effect of gut-training and feeding-challenge on this aspect of gastrointestinal status.

While these outcomes do not support the benefit of gut-training in terms of preserving gut integrity in response to exercise, they do, however, confirm the established minimum threshold for exercise stress that induces meaningful disturbances to gastrointestinal integrity within experimental designs and test EIGS and/or Ex-GIS prevention and management interventions. This has previously been shown to either be at least 2 h at 60% $\dot{V}{\rm O}_{2\max }$ in the heat (≥ 35 °C) or at least 3 h at 60% $\dot{V}{\rm O}_{2\max }$ in temperate conditions [25, 26, 70, 71]. Moreover, other proposed adaptations presented in previous gut-training narrative and opinion-based reviews include potential improvements in stomach volume and intestinal absorption capacity [30–32], which would be reflected more in terms of function or symptoms. Thus, if practitioners or athletes are aiming to reduce the incidence and magnitude of intestinal injury through gut-training or feeding-challenge during exercise, current evidence shows its effect to have no performance or clinical relevance.

### Gastrointestinal Function

Exercise-associated changes in gastrointestinal function appear to be related to the overall stress load of an activity. Specifically, a dose–response relationship between gastric emptying and exercise intensity or duration has previously been demonstrated, wherein GER is slower during exercise at higher intensities or longer durations [72]. An increase in intra-gastric pressure would result from a slower GER and orocecal transit time (OCTT), which can be compounded with feeding, and would potentially lead to nutrient malabsorption and further exacerbation of Ex-GIS [40, 73, 74]. In terms of gastrointestinal function, two studies showed that GER did not improve over time with repetitive ingestion of a carbohydrate solution during cycling and running at 65–75% $\dot{V}{\rm O}_{2\max }$ [47, 48]. Other more specific or sensitive methods to assess gastrointestinal motility (e.g., fluoroscopic techniques, electrogastrography, and ingestible gas-sensing capsule) [75–78] may provide further insight. While measuring GER using gastric aspiration is a common technique, it is very invasive and difficult to implement in exercise studies. Comparisons between the two studies that measured GER are challenged by the differences in volume ingested, beverage composition (e.g., carbohydrate versus carbohydrate electrolyte solution), and drinking patterns employed. Osmolarity, acidity, fat and amino acid content, and volume are all known determinants of gastric emptying of liquids [40]. The well-established role of drink volume in maintaining intragastric volume and pressure to facilitate emptying [79] was demonstrated by the two included studies [47, 48]. Frequent ingestion of small volumes as opposed to a bolus amount maintains a constant intragastric pressure, regulates GER, and impacts stomach comfort and even performance overall [29, 79]. This pattern of drinking is also more fitting for athletes in a race who rely on aid stations for food and fluids that are usually located several kilometers apart.

Intestinal nutrient transporters may play a role in preventing malabsorption and subsequent Ex-GIS. Carbohydrate malabsorption was improved with the repetitive ingestion of carbohydrate during exercise at 30 g every 20 min for 2 weeks [5, 49] with breath H_2_ responses observed to be positively correlated with gut discomfort and severity of Ex-GIS. One study did not observe any significant changes with breath H_2_ concentration post-intervention between dietary groups [28], likely as a result of the large individual variability within participants and/or the lower exercise stress load compared with previous 3-h experimental protocols [5, 49]. In this study [28], it is important to note that compared with the group consuming 30 g CHO/h, the group consuming carbohydrate at higher rates (highest tolerable rates between 70 and 100 g CHO/h) had more athletes with breath H_2_ concentrations greater than the clinical threshold for malabsorption (maximum-carbohydrate group: 56% at pre-intervention and 44% at post-intervention; control: 30% at pre- and post-intervention). This may demonstrate the oversaturation of intestinal nutrient transporters leading to incomplete absorption of carbohydrates [80]. Despite the proposed upregulation of intestinal carbohydrate transporters (e.g. GLUT 5, SGLT1, and/or GLUT 2) that can occur with gut-training, this has yet to be measured in any human study. With the use of non-metabolizable glucose analogs such as D-xylose and 3-O-methyl-D-glucose (3MG) in a solution, urinary excretion can be measured in order to determine passive and active intestinal absorption [24]. It can only be speculated that the reduction of breath H_2_ levels demonstrated by the two studies [5, 49] was due to an increase in absorption rate and capacity, leading to less residual carbohydrate in the gut that would translate to a reduction of osmotic load. It is important to also consider the role of individual differences in these aspects of gastrointestinal function. Furthermore, there are issues related to the reproducibility of breath H_2_ testing associated with various factors, such as confounder control (e.g., exercise, diet, and gastrointestinal microbial composition), interpretation for patients with low breath H_2_, low sensitivity and specificity, assumptions on the site of H_2_ production, and differences in testing protocol [81], and as such, the use of other gastrointestinal functional measures is warranted in future studies. Overall, considering the limited and fragmented methodologies within experimental designs, it appears gut-training and feeding-challenge during exercise does not facilitate improvements in GER. However, it is important to note that, to date, no study has utilized an array of gastrointestinal functional measurements using a well-defined gut-training protocol to thoroughly assess whether repetitive gastrointestinal challenge can alter functional aspects of the gastrointestinal tract. Nevertheless, it can be stated that gut-training could be beneficial in minimizing the risk of further exacerbating Ex-GIS among athletes who have malabsorption issues caused by high nutrient loads during exercise.

### Systemic Responses

Following exercise-associated injury to intestinal epithelial cells, epithelial hyperpermeability, and translocation of potential luminal-originating pathogenic microbial agents into the systemic circulation, an inflammatory cascade can be triggered at a local and systemic level [82]. As a result of exercise stress that leads to perturbations of intestinal epithelial integrity, the classical systemic inflammatory cytokine response includes no to mild increases in pro-inflammatory cytokines (e.g., IL-1β and TNFα) and modest increases in inflammatory response cytokines (i.e., IL-6 and IL-8) peaking immediately after exercise, and substantial elevations in anti-inflammatory cytokines peaking 1–2 h post-exercise, with the magnitude generally proportional to the exertional stress load [83–86]. Only in cases of extreme exertion and exertional heat stress exposure (e.g., ultra-endurance competition) does the systemic inflammatory cytokine profile mirror more closely a sepsis characteristic profile [87, 88]. Based on the findings of this current SLR, the effect of gut-training and feeding-challenge around exercise on systemic immune response remains unclear. One study found no significant difference in cytokine response between groups with varied daily carbohydrate intakes undertaking a 28-day nutrition and exercise intervention [50], while another study found that the high-carbohydrate group (24 g before and 60 g/h during exercise) had a lower pro-inflammatory cytokine response (e.g., IL-1α and IL-1β) compared with the lower-carbohydrate group (2.4 g before and 20 g/h during exercise) after 8 days of intensified training [51]. Interpreting outcomes of systemic immune response alongside endotoxin concentration and gastrointestinal symptoms would provide further insight on the effect of gut-training and feeding-challenge; however, these were not included as outcomes in these studies. Both studies were primarily interested in carbohydrate intake on immune markers, which has previously been shown to attenuate cytokine response to exercise in running and cycling models [89–91]. The lack of changes in systemic immune response observed in the study by Cox et al. [50] can be attributed to the amount of dietary carbohydrate intake being compared, which in general, was both adequate and within the range of the carbohydrate recommendations for athletic populations (~ 35% and ~ 60% of daily energy requirements) [2], and/or to the lack of exertional stress load to induce a substantial exercise-associated systemic inflammatory cytokine response [83]. Compare this with a similar study that investigated carbohydrate intakes with a larger difference (< 10% and 70% of daily energy requirements), which observed a greater reduction in IL-6, IL-10, and IL-1ra response to exercise in the higher carbohydrate group after a 3-day dietary intervention [92]. Furthermore, in the study by Svendsen et al. [51], it is important to note that the significant attenuation in pro-inflammatory cytokine response in the higher-carbohydrate group compared with the lower-carbohydrate group was assessed at rest following 8 days of intensified training. The differences in the exercise protocol and the duration and specifics of the nutrition interventions in the two studies make it challenging to compare and come to a generalization. Overall, the benefits of gut-training or feeding-challenge during exercise on systemic immune response warrant further investigation. Thus, if the aim is to improve cytokine balance by further increasing carbohydrate intake alone through additional feeding around exercise, it appears to be ineffective.

### Exercise-Associated Gastrointestinal Symptoms (Ex-GIS)

Ex-GIS is commonly reported amongst the active population, especially among those undertaking endurance-type exercise [11, 13–15, 53, 93–95]. This has major implications in terms of not only performance outcomes, but also nutritional intake and tolerance around exercise, as well as clinical implications. The two primary causal pathways associated with EIGS, namely the circulatory–gastrointestinal and the neuroendocrine–gastrointestinal pathways, underpin these symptoms [17, 52, 53]. Depending on the origin, symptoms can be classified into upper gastrointestinal (e.g., belching, stomach pain, regurgitation, heartburn, etc.), lower gastrointestinal (e.g., flatulence, lower abdominal bloating, urge to defecate, intestinal pain, diarrhea, etc.), or other gastrointestinal-related symptoms (e.g., nausea, dizziness, acute transient abdominal pain) [96] which is important in diagnosing and determining management approaches [81]. Several established factors may also predispose an individual to Ex-GIS, such as exercise intensity [23, 24, 73], duration [61, 97], and modality [53, 98], biological sex [99, 100], environmental temperature [22, 26], nutrition intake before and/or during exercise [13, 14, 93], hydration status before and during exercise [101], and/or history of Ex-GIS [5, 102].

Results from the current SLR showed improvements in gastrointestinal discomfort and Ex-GIS with gut-training or feeding-challenge during and after exercise, but only in some studies. Significant reductions in gut discomfort were observed after 2 weeks of gut-training with carbohydrate [5, 49] and repeated ingestion of a carbohydrate solution across six trials [48] during running. Furthermore, in one study that evaluated carbohydrate (1.2 g/kg/h) and carbohydrate and protein (0.87 g/kg/h and 0.33 g/kg/h, respectively) ingestion during Australian Rules Football matches (90 min) and training (60-min ball skills session), no significant differences in median gut discomfort scores were observed based on a generic assessment tool [46]. The exertional stress loads described in this study are not synonymous with EIGS and, along with the use of a broad and non-specific Ex-GIS assessment tool, may have likely impacted the accuracy of the results [55, 81]. It is also important to consider that ingestion of protein during prolonged strenuous exercise in the heat has been associated with high incidence and severity of Ex-GIS [15], exacerbated gut discomfort, and total and upper Ex-GIS when using a validated and reliability checked Ex-GIS assessment tool [5]. Total, lower and upper gastrointestinal symptoms were improved with 2 weeks of gut-training [5, 49] but not in the study by King et al. [28]. This is likely attributable to the study population, and Ex-GIS outcomes between highly trained endurance athletes accustomed to consuming food and fluid during exercise versus recreational endurance athletes who are less accustomed to feeding during exercise. This observation is reinforced by the data presented in Costa et al. [5], whereby higher fitness status and being accustomed to feeding during exercise resulted in lower changes in gut-training outcomes, which is consistent with King et al. [28]. Moreover, a significant reduction in nausea in response to a 3-h feeding-challenge was reported after 2 weeks of gut-training [5], but improvements related to feeding-tolerance variables (e.g., thirst, fullness, tolerance to food/drink, etc.) with the gut-training protocol were not observed [49].

As previously mentioned, among these studies, both validated and unvalidated tools were used to collect data on Ex-GIS, which could have led to erroneous or misleading findings. Due to the highly variable measurement tools used to assess gastrointestinal symptoms, comparison between studies is difficult; this may also explain the lack of cohesiveness in the effect of gut-training or feeding-challenge observed on Ex-GIS. The exercise-specific mVAS [71] was recently developed based on the tool routinely used for irritable bowel syndrome diagnosis in gastroenterology in clinical settings [103]. It was created to address exercise-specific situations related to gastrointestinal issues experienced by athletes. This tool was used in only one of the included studies in this review [28], but an earlier version was used in two included studies [5, 49] wherein study participants were educated on how to complete the assessment tool. Such assessment tool standardization should be used in future studies investigating gut-training or feeding-challenges during exercise to avoid erroneous data collection.

Based on the Ex-GIS finding, the proposed notion of the ability of the stomach to accommodate larger volumes of food or fluid after repeated exposures is supported by the results from Costa et al. [5], Miall et al. [49], and Lambert et al. [48], and further highlights this aspect of the ‘trainability’ of the gut. In the study by Lee et al. [46], it would be expected that the carbohydrate with protein group would experience greater gut discomfort or incidence and severity of Ex-GIS. Proteins are complex molecules that require a longer time to be broken down compared with the more easily digested simple sugars [104]. This has been demonstrated in a previous study among endurance athletes wherein the ingestion of 15 g of whey protein before and during exercise (every 20 min) resulted in greater Ex-GIS despite its beneficial effects on gut integrity [9]. Alternative measures of gastrointestinal function are warranted to further investigate this, as Ex-GIS improvements could be attributed to a reduction in intragastric pressure resulting from increased gastric capacity and motility [40, 72, 73]. Additionally, it could also be postulated that enhanced absorption of carbohydrate led to improvements in observed lower gastrointestinal symptoms, specifically in the studies by Costa et al. [5] and Miall et al. [49], as evidenced by reductions in breath H_2_ concentrations in response to carbohydrate feeding during exercise [35, 105, 106]. On the other hand, contrasting findings on gastrointestinal symptoms may be attributed to the triggering of the ileal break by gastrointestinal components such as dietary fat, protein, and carbohydrate causing a decrease in gastric emptying and gastrointestinal motility [39], and potentially contributing to reduced feeding tolerance and increased gastrointestinal symptoms.

Overall, the major differences in athlete populations, assessment methods, exercise protocol, and nutrition intervention when symptoms were measured make comparisons challenging between studies. It is important to note that the study by Lee et al. [46] was among younger Australian Rules Football athletes and not endurance athletes. Despite the endurance features of football training or matches, it is difficult to compare these findings from studies with a prolonged sub-maximal exercise model given that the matches had a shorter duration (~ 90 min) with breaks and were intermittent in nature. There was also variability in the level of athletes (e.g., junior, recreationally competitive, trained, and elite) included in this review, which could partially explain the differences observed in terms of Ex-GIS. Although positive effects of gut-training on total, upper, and lower GIS were consistent in two studies [5, 49], there were inconsistent and contrasting findings from another study [28], which can be largely explained by the differences in the assessment method, intervention structure, and study population. Specifically, the studies by King et al. [28] included elite-level race walkers and runners who are most likely already used to consuming a large amount of carbohydrate around training and competition, which has implications on the effects of gut-training. This is in comparison with the studies by Costa et al. [5] and Miall et al. [49], which included recreationally competitive distance runners who were not accustomed to consuming large volumes of carbohydrates around exercise, in which the authors found improvements in Ex-GIS outcomes. In addition, a major difference in study design and intervention can be noted between the studies by King et al. [28] and those by Costa et al. [5] and Miall et al. [49], wherein the latter studies mimicked the same nutrition conditions during training and trial days (30 g/h every 20 min) and had a control placebo group. In contrast, the studies by King et al. [28] had a control group that consumed 30 g/h during both gut-training and trial days. In the maximum carbohydrate group, gut-training was done using a range of 60–90 g/h but during trial days it was set at 90 g/h for the race walkers and at highest tolerable rates between 70–100 g/h among the runners. These key differences make these two studies non-comparable even with the same duration of the intervention and the slightly similar Ex-GIS assessment tool used. Nonetheless, there is some agreement among the findings that suggest improvements in gut discomfort and upper GIS as a result of gut-training or feeding-challenges around exercise. As such, the results suggest that gut-training and feeding-challenge before and during exercise would help athletes improve gastrointestinal comfort and could potentially reduce the risk of Ex-GIS when consuming food and fluid around exercise.

### Links to Exercise Performance

Physiological adaptations to gut-training and feeding-challenges around exercise may result in improved exercise performance. Blood glucose availability and subsequent uptake of skeletal muscles for oxidation would be directly influenced by gastrointestinal function during exercise. Moreover, tolerance to feeding and the incidence and severity of Ex-GIS would greatly impact the nutritional intake of athletes during exercise, which may hamper performance.

Among the included studies, two studies had outcomes related to glucose availability and/or muscle fuel kinetics. In the study by Costa et al. [5], post-intervention blood glucose concentration after 2 h of steady-state running at 60% $\dot{V}{\rm O}_{2\max }$ followed by a 1-h distance test was significantly higher in the carbohydrate-supplement group (7.2 mmol/L) compared with the carbohydrate-food (6.1 mmol/L) and placebo (6.2 mmol/L) groups. In addition, after gut-training, blood glucose concentration was also significantly higher in the carbohydrate-supplement group during the 2-h run at the 90-min timepoint compared with the placebo group (6.6 vs 5.9 mmol/L, respectively). It can be speculated that these findings are likely associated with changes in intestinal sugar transporters (e.g., increased activity), as a flatline result in the H_2_ breath test was observed after gut-training which was previously increased in the pre-gut-training trial.

In contrast, in the Cox et al. study [50], there were no significant differences between the moderate-carbohydrate group and higher-carbohydrate group in pre- (4.9 and 4.7 mmol/L, respectively) and post-exercise (4.7 and 4.5 mmol/L, respectively) plasma glucose concentration before the intervention. Likewise, post-intervention plasma glucose concentrations of the moderate-carbohydrate group and higher-carbohydrate group before (4.7 vs 4.6 mmol/L, respectively) and after exercise (4.6 vs 5.0 mmol/L, respectively) were not significantly different. The exercise protocol in this study was 100 min of cycling at 70% $\dot{V}{\rm O}_{2\max }$ followed by a time trial at a pre-determined workload of 7 kJ/kg. In the study by Houmard et al. [47], blood glucose level after 1 h of cycling at 75% $\dot{V}{\rm O}_{2\max }$ was significantly higher in the carbohydrate trial (5.6 mmol) compared with the water trial (4.4 mmol). No significant difference in post-exercise blood glucose level was observed between the running with carbohydrate trial (5.2 mmol) and the running with water trial (4.7 mmol). Muscle fuel kinetics was only assessed in the study by Costa et al. [5] wherein total fat and carbohydrate oxidation during 2-h steady-state running at pre- and post-intervention were similar between groups. Exogenous carbohydrate oxidation for the Cox et al. study [41] was increased only in the higher-carbohydrate group but with no apparent improvement in exercise performance compared with the moderate-carbohydrate group. As such, it appears that repeatedly challenging the gut with a high carbohydrate load (e.g., 90 g/h) during training or chronic dietary carbohydrate supplementation (e.g., 8.5 g/kg for 28 days) has implications on the regulation of blood glucose availability or exogenous carbohydrate oxidation, which can be a function of decreased malabsorption or potentially intestinal transport improvements.

In terms of performance, the carbohydrate-supplement and carbohydrate-food gut-training groups in the Costa et al. [5] study and the carbohydrate group in the Miall et al. study [49] covered more distance after gut-training compared with the placebo group. In the Costa et al. [5] study, the greater improvement observed in the carbohydrate-supplement group is likely explained by the increased glucose availability because of less malabsorption, plus a decrease in lower GIS. In contrast, performance improvements in the carbohydrate-food group may only be due to the reduction in lower GIS. Similarly, performance improvements in the carbohydrate group in the Miall et al. study [49] could be largely explained by the reduction in GIS after gut-training. These findings highlight that onset of GIS impacts the ability of an individual to maintain the exercise workload, which has major implications on performance. In contrast, no performance effects were observed among the runners in the King et al. [28] study, which can be attributed to the fact that these were elite athletes that were already performing at a high level and improvements in GIS were also not observed. In the Cox et al. [41] study, despite an increase in exogenous carbohydrate oxidation in the higher-carbohydrate group compared with the moderate-carbohydrate group, this did not translate into any exercise performance improvements. Overall, the gut’s role in regulating blood glucose availability, and the occurrence of GIS, could potentially influence exercise performance, with training status impacting the magnitude of benefits.

### Risk of Bias

Risk-of-bias assessment is important in determining the potential influence of study features or conduct on results. Based on the results of the assessment of the included studies, three studies [5, 28, 47] were assessed as having ‘some concerns’ in terms of overall risk of bias. This was a result of not explicitly stating their blinding and randomization procedures, or it was not possible to entirely blind participants and/or the researchers to the intervention assignments. The majority of included studies had an overall low risk of bias. Interestingly, based on the interventions implemented in these studies, it is demonstrated that a standard gut-training protocol does not yet exist. While some of the studies that were reviewed incorporated a well-defined gut-training strategy (e.g., specific dose, frequency, and duration), this was only implicitly done in the other studies (e.g., as a result of the study design or intervention duration) through feeding-challenges around exercise. To date, no study has compared different ways to train the gut through modifying factors such as nutrient composition of the food used to challenge the gut or varying the duration and frequency of gut-training or feeding-challenges. The high variability in the gut-training and feeding-challenge protocols conducted in the included studies makes it difficult to determine the ideal amount of time required to implement this type of targeted nutritional training to see benefits. The 2-week gut-training protocol originally tested by Costa et al. [5] was employed in four studies, but due to key differences in population and testing methods, generalizations cannot be made in terms of the outcomes.

### Limitations and Future Directions

The authors recognize that there are several limitations within this current SLR. First, there is a potential for systematic bias due to use of language restrictions to English only in the search process. Furthermore, a majority of the participants in the included studies were males and given the sex-specific differences in physiological parameters, findings may not be representative of the responses among female athletes. This is particularly relevant for gastrointestinal status outcomes, especially if menstrual cycle phase is not standardized during trials [107].

Future directions for research in this field should explore elucidating the mechanisms behind the proposed adaptations via including more sensitive/specific markers to assess gastrointestinal integrity (e.g., non-metabolizable sugar probes, circulating concentration of LPS, sCD14, Ig and EndoCAb), function and transit times (e.g., lactulose or carbon-13 breath tests, gas-sensing capsule), and intestinal absorption (e.g., non-metabolizable glucose analogs such as D-xylose and 3MG). The use of a standardized and validated tool such as the mVAS will enable better comparison between studies and elucidate the effects of gut-training and feeding-challenges on Ex-GIS. These suggested measures could be part of a standard test battery that is currently warranted to assess gastrointestinal status and EIGS-related outcomes [65]. Moreover, unexplored areas also include the potential role of the microbiota and microbiota-derived metabolites (e.g., short-chain fatty acids [SCFA]) in facilitating the changes observed with gut-training. Investigating other endurance exercise stress models (i.e., combining running and cycling to mimic long-course triathlon, modifying duration or intensity of a running or cycling protocol) would also provide further insight on factors such as exercise modality, duration, and intensity and if thresholds exist in relation to the effects of gut-training. Lastly, to develop a well-defined strategy, there is a need to investigate varying gut-training or feeding-challenge protocols to assess the best way to reap the benefits from this type of targeted nutritional training. Specifically, in the area of gut-training, the existing studies have only investigated training the gut with carbohydrates, when in reality, some athletes consume carbohydrate-rich food that contains other nutrients (e.g., fat and protein) during races. Avenues for future research would be investigating the impact of different aspects of the nutrition intervention (e.g., macronutrient composition and density, fermentable carbohydrate content, physical form of the food, volume), as well as the timing, frequency, and duration of the protocol. As an example, in terms of duration, it is currently unknown if benefits would be observed with a shorter protocol (e.g., < 2 weeks) or if a longer protocol would lead to greater benefits. Ultimately, it is important that future studies investigating variations of gut-training or feeding-challenge during exercise use standardized assessment tools and choose comparable exercise stress models in order to build on current knowledge.

## Conclusions

The potential adaptations from gut-training and feeding-challenge around exercise may offer advantages in terms of supporting delivery of fuel to the muscles, managing Ex-GIS, and preventing clinical implications associated with EIGS. Although very limited literature is available on the topic of gut training and feeding-challenge around exercise and several quality issues were identified among the included studies, it appears that gut-training or feeding-challenge around exercise improves gut discomfort during exercise, may reduce carbohydrate malabsorption in response to feeding during exercise, and reduces the incidence and severity of Ex-GIS, predominantly upper-GIS types. These gastrointestinal objective and subjective improvements have been linked with enhanced glucose availability and direct exercise performance (i.e., distance test). No study to date has investigated the potential mechanisms to explain these observed benefits. Additional research is also needed to identify the best approach in training the gut. The proposed adaptations from the repetitive exposure of the gut to similar race-day nutrition conditions, in theory, would be highly beneficial to athletes, not just for performance, but also in the prevention of clinical issues warranting medical management.

## Supplementary Information

Below is the link to the electronic supplementary material.

Supplementary file1 (DOCX 17 KB)
