# Supplementary material for: The Effect of Gut-Training and Feeding-Challenge on Markers of Gastrointestinal Status in Response to Endurance Exercise: A Systematic Literature Review
Source: Sports Med. 2023 Apr 15;53(6):1175–200. doi: 10.1007/s40279-023-01841-0 (PMC10185635; doi:10.1007/s40279-023-01841-0)
Supplement: Supplementary file 1 — Supplementary file1 (DOCX 17 KB) [file 40279_2023_1841_MOESM1_ESM.docx]

**Systematic Literature Review: The Effect of Gut-training and Feeding Challenges on Markers of Gastrointestinal Status in Response to Exercise**

**Isabel G. Martinez^1^, Alice S. Mika^1^, Jessica R. Biesiekierski^1^, Ricardo J.S. Costa^1*^**

^1^Department of Nutrition, Dietetics and Food, Monash University, Notting Hill, Australia

**Corresponding author:**

Ricardo J.S. Costa: Monash University, Department of Nutrition & Dietetics, Level 1, 264 Ferntree Gully Road, Notting Hill, 3168, Victoria, Australia. Telephone: 00 61 3 99024270. Email: ricardo.costa@monash.edu

Supplementary Information 1. Search strategy used for the systematic literature review on effect of gut-training on markers of gastrointestinal status in response to exercise

| **Field One (combine with OR):**  **Population** |  | **Field Two (combine with OR): Intervention** |  | **Field Three (combine with OR): Outcome** |
| --- | --- | --- | --- | --- |
| Keywords: Endurance exercise, Endurance sport, Endurance athlete, Endurance training, Run*, Cycling, cyclist*, Triathlon, Triathlete, Distance run*, Prolonged exercise  MeSH headings: Exercise/me, ph [Metabolism, Physiology], Athletes/ Running/ or Marathon Running/, Bicycling/ | AND | Keywords: Gut training, Gut challenge, Training the gut, Fluid tolerance, Exogenous glucose, High carbohydrate availability, carbohydrate supplementation, Carbohydrate feeding during exercise, Carbohydrate ingestion during exercise, Repeated drinking during exercise  MeSH headings: Adaptation, physiological/, dietary carbohydrates/ad, Dietary sugars/ad, Drinking/ph [Physiology], Sports Nutritional Physiological Phenomena/ph [Physiology], Dietary supplements/, Diet, carbohydrate loading | AND | Keywords: gastrointestinal injury, gastrointestinal damage, gastrointestinal permeability, Intestinal fatty acid binding protein, I-FABP, mucosal barrier, tight junction, zonulin, claudin, dual sugar tests, lactulose, rhamnose, urinary sugars, endotoxemia, lipopolysaccharide*, LPS, LAL, gram negative bacteria, sCD14, LBP, cytokine*, gastrointestinal function, gastrointestinal motility, gastrointestinal emptying, gastrointestinal malabsorption, orocecal transit time, OCTT, electrogastrogram, EGG, gastric aspiration, ^13^C acetate breath test, radioisotope scanning,  hydrogen breath test, methane breath test, microbiota, microbial composition, SCFA, short chain fatty acids*, gastrointestinal symptom*, gastrointestinal comfort, gastrointestinal tolerance  MeSH headings: Gastrointestinal tract/, Gastrointestinal motility/, gastric emptying/, gastrointestinal transit/, Microbiota/ |

** Used to retrieve unlimited suffix variations*
